# Supplementary material for: Smartphone-Guided Educational Counseling and Self-Help for Chronic Tinnitus
Source: J Clin Med. 2022 Mar 25;11(7):1825. doi: 10.3390/jcm11071825 (PMC8999555; doi:10.3390/jcm11071825)
Supplement: Supplementary file 1 [file jcm-11-01825-s001.zip › jcm-1597828-supplementary.pdf]

Supplemental Material

S1. Supplemental Material: Example of daily tips
Two Examples of tinnitus tips are displayed. A screenshot of the app is given in the figure. The original German tip and it's translations are provided in the table below.

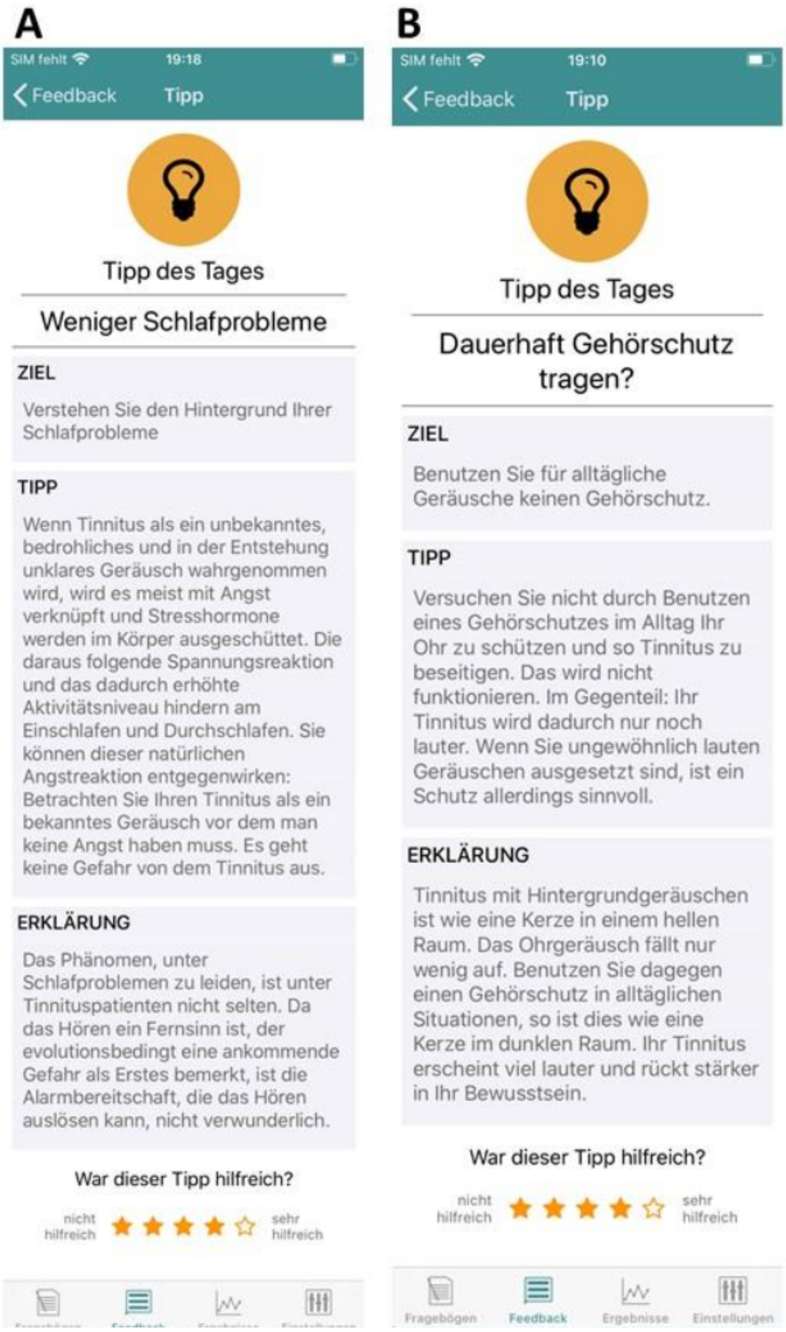

	**German original**	**English translation**

Example A	Verstehen Sie den Hintergrund Ihrer Schlafprobleme.<br>ZIEL: Verstehen Sie den Hintergrund Ihrer Schlafprobleme.<br>TIPP: Wenn Tinnitus als ein unbekanntes, bedrohliches und in der Entstehung unklares Geräusch wahrgenommen wird, wird es meist mit Angst verknüpft und Stresshormone werden im Körper ausgeschüttet. Die daraus folgende Spannungsreaktion und das dadurch erhöhte Aktivitätsniveau hindern am Einschlafen und Durchschlafen. Wirken Sie dieser natürlichen Angstreaktion entgegen, indem Sie durch beruhigende Information den Tinnitus in ein richtiges Licht rücken.<br>ERKLÄRUNG: Das Phänomen, unter Schlafproblemen zu leiden, ist unter Tinnituspatienten nicht selten. Da das Hören ein Fernsinn ist, der evolutionsbedingt eine ankommende Gefahr als Erstes bemerkt, ist die Alarmbereitschaft, die das Hören auslösen kann, nicht verwunderlich.	Understand the background of your sleep problems.<br>OBJECTIVE: Understand the background of your sleep problems.<br>TIP: When tinnitus is perceived as an unknown, threatening sound that is unclear in origin, it is usually associated with anxiety and stress hormones are released in the body. The resulting tension reaction and the resulting increased level of activity prevent us from falling asleep and sleeping through the night. Counteract this natural fear reaction by using calming information to put the tinnitus in perspective.<br>EXPLANATION: The phenomenon of suffering from sleep problems is not uncommon among tinnitus patients. Since hearing is a remote sense that evolutionarily notices an approaching danger first, the alertness that the hearing sense can trigger is not surprising.
Example B	ZIEL: Benutzen Sie für alltägliche Geräusche keinen Gehörschutz.<br>TIPP: Versuchen Sie nicht durch Benutzen eines Gehörschutzes im Alltag Ihr Ohr zu schützen und so Tinnitus zu beseitigen. Im Gegenteil: Ihr Tinnitus wird dadurch nur noch lauter. Wenn Sie ungewöhnlich lauten Geräuschen ausgesetzt sind, ist ein Schutz allerdings sinnvoll.<br>ERKLÄRUNG: Tinnitus mit Hintergrundgeräuschen ist wie eine Kerze in einem hellen Raum. Das Ohrgeräusch fällt nur wenig auf. Benutzen Sie dagegen einen Gehörschutz in alltäglichen Situationen, so ist dies wie eine Kerze im dunklen Raum. Ihr Tinnitus erscheint viel lauter und rückt stärker in Ihr Bewusstsein.	OBJECTIVE: Do not use hearing protection for everyday sounds.<br>TIP: Do not try to protect your ear by using hearing protection in everyday life and thus eliminate tinnitus. On the contrary, it will only make your tinnitus louder. However, if you are exposed to unusually loud sounds, protection is useful.<br>EXPLANATION: Tinnitus with background noise is like a candle in a bright room. The ear noise is only slightly noticeable. If, on the other hand, you use hearing protection in everyday situations, it is like a candle in a dark room. Your tinnitus appears much louder and becomes more noticeable.

Supplemental Material

S2. Supplemental Material: Screenshot of the Ecological Momentary Assessment
A screenshot of the Ecological Momentary Assessment is given in the figure. The original German questionnaire and it's translations is provided in the table below.

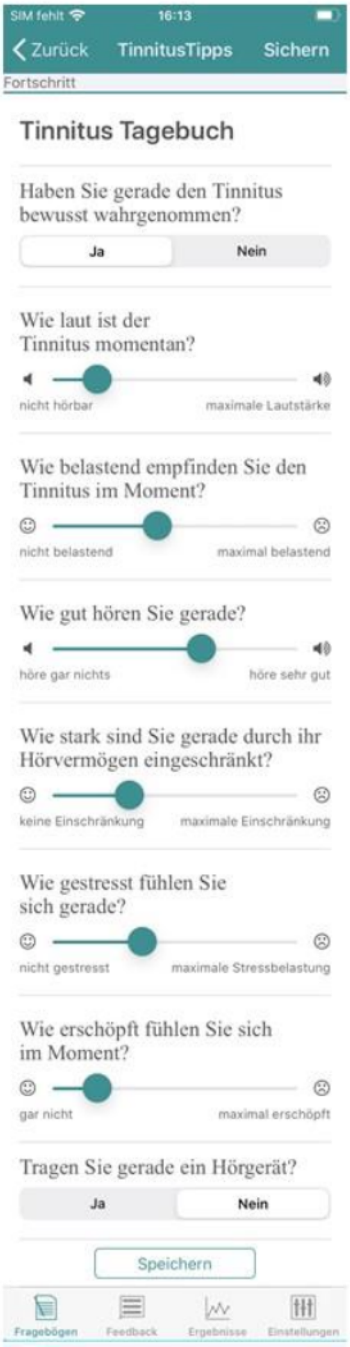

	German original	English translation
Question 1	Haben Sie gerade den Tinnitus bewusst wahrgenommen?	Did you just consciously notice the tinnitus?
Question 2	Wie laut ist der Tinnitus momentan?	How loud is the tinnitus right now?
Question 3	Wie belastend empfinden Sie den Tinnitus im Moment?	How burdensome is the tinnitus at the moment?
Question 4	Wie gut hören Sie gerade?	How well are you hearing right now?
Question 5	Wie stark sind Sie gerade durch ihr Hörvermögen eingeschränkt?	How limited are you by your hearing ability right now?
Question 6	Wie gestresst fühlen Sie sich gerade?	How stressed do you feel right now?
Question 7	Wie erschöpft fühlen Sie sich im Moment?	How exhausted do you feel at the moment?
Question 8	Tragen Sie gerade ein Hörgerät?	Are you currently wearing a hearing aid?

Supplemental Material 3:
English Translation of the Tinnitus Empowerment Scale (TES)

Below are some statements people sometimes make about their tinnitus, which may be more or less true in your personal case.

All statements are about now - please indicate how strongly these statements apply to you ***currently***.

	absolutely correct	rather correct	draw	rather wrong	completely wrong
I am generally well informed about tinnitus.					
When my tinnitus is louder than normal, I know what to do to make it softer again.					
I am convinced that I will find solutions for my tinnitus.					
At my next doctor's appointment, I know exactly what questions I want to ask.					
I can prevent my tinnitus from getting worse.					
I know the cause of my tinnitus.					
I can mostly help myself with my tinnitus.					
I know the influencing factors that make my tinnitus louder.					
I know the influencing factors that make my tinnitus softer.					
I read scientific articles about tinnitus.					
I am able to influence the tinnitus through my behavior.					
I can independently judge which information about tinnitus (e.g. on the Internet or in advertising) is true or false.					
I am aware that my behavior can have an impact on my tinnitus.					
Even when I'm traveling, I can make sure my tinnitus doesn't get louder.					
I can cope well with my tinnitus.					
I can talk openly with other people about my tinnitus.					
